# Definition of High-Risk Motion Patterns for Female ACL Injury Based on Football-Specific Field Data: A Wearable Sensors Plus Data Mining Approach

**DOI:** 10.3390/s23042176

**Published:** 2023-02-15

**Authors:** Stefano Di Paolo, Eline M. Nijmeijer, Laura Bragonzoni, Alli Gokeler, Anne Benjaminse

**Affiliations:** 1 Department for Life Quality Studies, University of Bologna, 40136 Bologna, Italy; 2 Department of Human Movement Sciences, University Medical Center Groningen, University of Groningen, 9713 AV Groningen, The Netherlands; 3 Exercise and Neuroscience Unit, Department Exercise & Health, Faculty of Science, University of Paderborn, 33098 Paderborn, Germany; 4 Amsterdam Collaboration for Health and Safety in Sports, Department of Public and Occupational Health, Amsterdam Movement Sciences, VU University Medical Center, 1081 HZ Amsterdam, The Netherlands; 5 Faculty of Health, Amsterdam University of Applied Sciences, 1091 GC Amsterdam, The Netherlands; 6 School of Sport Studies, Hanze University Groningen, 9747 AS Groningen, The Netherlands

**Keywords:** ACL, football, injury prevention, agility, wearable sensors, agglomerative clustering, ecological dynamics

## Abstract

The aim of the present study was to investigate if the presence of anterior cruciate ligament (ACL) injury risk factors depicted in the laboratory would reflect at-risk patterns in football-specific field data. Twenty-four female footballers (14.9 ± 0.9 year) performed unanticipated cutting maneuvers in a laboratory setting and on the football pitch during football-specific exercises (F-EX) and games (F-GAME). Knee joint moments were collected in the laboratory and grouped using hierarchical agglomerative clustering. The clusters were used to investigate the kinematics collected on field through wearable sensors. Three clusters emerged: Cluster 1 presented the lowest knee moments; Cluster 2 presented high knee extension but low knee abduction and rotation moments; Cluster 3 presented the highest knee abduction, extension, and external rotation moments. In F-EX, greater knee abduction angles were found in Cluster 2 and 3 compared to Cluster 1 (*p* = 0.007). Cluster 2 showed the lowest knee and hip flexion angles (*p* < 0.013). Cluster 3 showed the greatest hip external rotation angles (*p* = 0.006). In F-GAME, Cluster 3 presented the greatest knee external rotation and lowest knee flexion angles (*p* = 0.003). Clinically relevant differences towards ACL injury identified in the laboratory reflected at-risk patterns only in part when cutting on the field: in the field, low-risk players exhibited similar kinematic patterns as the high-risk players. Therefore, in-lab injury risk screening may lack ecological validity.

## 1. Introduction

Anterior cruciate ligament (ACL) rupture is one of the most devastating musculoskeletal injuries in sport population and has both short- and long-term consequences for an athlete’s health and sport career [1,2]. In football (soccer, https://www.fifa.com/, accessed on 9 February 2023), non-contact ACL injuries in females typically occur during dynamic movements such as rapid deceleration or change of direction while pressing [3,4]). The actual injury is the result from a dynamically varying interaction among the player’s characteristics, the stimulus-rich environment and the desired actions [3,5,6].

Establishing ACL injury risk during cutting movements has traditionally been performed in relatively standardized laboratory environments [7,8]. This current paradigm inherently assumes that cutting biomechanics is independent of a sport-specific context. Laboratory results show increased hip and knee abduction moments during unanticipated cutting tasks [9] and in the presence of a defender [10] compared to anticipated ones. Even though these efforts provide insight into what adding sport-specific elements does to movement, the ecological validity can still be questioned [5,11,12,13].

As movement emerges per definition from the interaction with the environment [14], the information we receive from this constrained type of testing may not be valid. Therefore, we have recently advocated to assess players’ motion in their ecological environment [5]. This is because more and more evidence is emerging showing that how athletes move in a relatively standardized environment is still not so informative for the motor strategy they adopt in a sport-specific context [13]. For example, kinematic differences, i.e., lower sagittal plane knee range of motion during agility movements, has been shown on the field compared to in the laboratory [13]. These differences pertain to observed on-field ACL injury mechanisms [3,4] and prevention strategies.

Therefore, measuring movement in an ecological valid environment may potentially reshape the current definition of high-risk biomechanics according to real environment data instead of laboratory data only. Thus, to examine whether athletes have a risk profile for ACL injury, it is advised to examine how they move on the field, the place where the actual ACL injuries occur, i.e., preserve the athlete–environment relationship [5].

Thus far, studies either collected data from the laboratory or on the field. To the best of the authors’ knowledge, a cohort of athletes have not been tested both in the laboratory and on the field. Such an analysis may help reshaping the current definition of high-risk biomechanics in ACL injury prevention.

Thus, the aim of the present study was to investigate if the presence of ACL injury risk factors depicted in the laboratory would reflect at-risk patterns in football-specific field data in female footballers. It was hypothesized that clinically relevant differences toward ACL injury risk seen in the laboratory would only partially reflect risk patterns in the field.

## 2. Materials and Methods

### 2.1. Participants

All procedures were approved by the Medical Ethical Committee of the Blinded for submission (ID number: Blinded for submission). All players and their parents/legal guardians signed informed consent before inclusion. Twenty-four healthy female highly talented female football (soccer) players (mean age 14.9 ± 0.9 year, height 167.9 ± 4.8 cm, mass 56.4 ± 7.3 kg) were included. All players were signed to a highest or second highest level football team of the Blinded for submission. Players’ engagement consisted in four to five training sessions (average training session time: 75 min) and one official game per week. Players’ dominant leg was identified as the preferred leg to jump and land with. Twenty players were identified as right dominant. The power analysis revealed a minimum of 17 players to have a power of 0.80 considering a partial eta squared of 0.10 (medium effect) and an alpha of 0.05 (G*Power v3.1.9).

### 2.2. Data Collection

Data collected were held during the regular football season (September–December and February–May). For the laboratory task, anthropometric measures were collected for each player. Sixteen reflective markers were placed according to the Vicon Plug-in-Gait lower body model (Vicon Motion Systems, INC., Centennial, CO, USA). Five additional trunk markers were placed on the sternum, clavicle, C7, T10, and right scapula. A static calibration (T-pose on the force plates) was then performed according to manufacturer’s guidelines (Bertec Corporation, Columbus, OH, USA) [15]. A 100 Hz eight camera motion analysis system (Vicon Motion Systems, INC., Centennial, CO, USA), Vicon Nexus Software (version 2.7 Motions Systems, INC., Centennial, CO, USA), and two 1000 Hz force plates were used to capture trunk and lower body kinematics and vertical ground reaction force (vGRF) data. Previous research has shown high test-retest repeatability and good measurement accuracy of Vicon motion analysis [16,17].

Kinematic data of the on-field tasks were collected using Xsens MVN Analyze system (Xsens Technologies, Enschede, The Netherlands). The full description of this part of the data collection is described in our previous study [13].

### 2.3. Agility Task

The laboratory and on-field tasks to be performed by the players are described in our previous study [13]. In short, players executed unanticipated sidestep cutting movements in the laboratory and on-field either the same day or within a few days. Players used a 5-m approach run followed by a 1-foot landing with the dominant (kicking) leg and a 40–50° change in the direction followed by running through a gate 5 m away in the laboratory. Five trials of the task were collected. The task was a mirror exercise in which the player had to respond as quick and accurate as possible to unanticipated changes of direction by a buddy.

On-field tasks were recorded during regular training sessions. Agility movements were divided into two conditions: exercise (F-EX) and game (F-GAME). The F-EX included all the most frequent football-specific elements: single and double leg jumping and landing, running, cutting, deceleration, and passing. Unexpected elements including the presence of the ball and/or an opponent were always included (e.g., anticipate a defender while changing direction). The F-GAME consisted in a training match (11 vs. 11 players) performed at the end of the training session [13].

### 2.4. Data Processing and Statistical Analysis

Laboratory data of the knee in all three directions were extracted and filtered with a fourth order-zero lag Butterworth low-pass filter at 10 Hz. The external knee moments were normalized to body mass. The joint kinematics collected on the field for knee, hip, ankle, and pelvis in all three directions were extracted from the Xsens MVN Analyze 2020.0.1 software suite (Xsens Technologies, Enschede, The Netherlands) and further processed in a customized script in MATLAB (The MathWorks, Natick, MA, USA). Joint angles were defined using the Euler sequence ZXY.

For the field, unanticipated sidestep cuts between 30° and 60° were isolated through visual inspection of the Center of Mass trajectory [18]. The ultimate foot contact before the change of direction was extracted and data were normalized from 50 ms prior to the initial contact (0%) to 25 ms after the toe-off (100%) [4,19]. The initial contact (IC) and the toe-off (TO) were found on average at 15.5% and 93.5% of the overall time window, respectively. The cut speed of each trial was computed through the Center of Mass velocity parameter. Overall, 280 valid trials (88 for the laboratory—LAB, 111 F-EX, and 81 F-GAME) were included in the final analysis.

Players were clustered according to the analysis of knee kinetics collected in the laboratory environment. The knee kinetics were used to be coherent with previous prospective studies assessing the ACL injury risk in football players [20,21]. The discrete wavelet transform with the Haar wavelet was used to decompose the signals and the hierarchical agglomerative clustering based on Ward’s linkage method which was used to group together the trials with the most similar features [22,23,24]. The clusters were then used to investigate the data collected on the field through the one-way ANOVA with hierarchical two-level random effect model in Statistical Parametric Mapping 1D (SPM1D). Players were included in the model as a random effect. The experimental flow chart has been summarized in Supplementary Materials.

## 3. Results

### 3.1. Laboratory-Based Clustering

**Figure 1:**
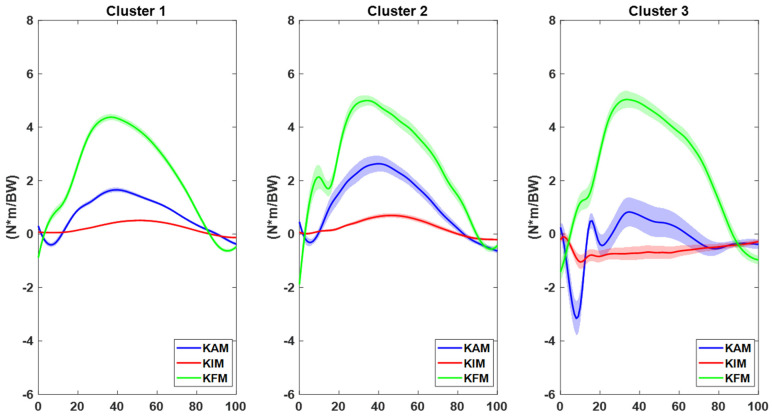
Knee kinetics clusters based on Haar wavelet collected in laboratory. Data are normalized over the stance phase of the change of direction and are presented as mean (solid lines) and standard deviation (shadow). Abbreviations: KAM = knee abduction moment (blue, negative: abduction); KIM = knee internal rotation moment (red, negative: external); KFM = knee flexion moment (green, negative: extension).

Three clusters emerged from the Haar wavelet analysis (Figure 1).

Cluster 1 included 61 trials (13 players) and presented the lowest peak knee moments at IC in frontal, sagittal, and transverse planes. Players in this cluster were thus considered at “low-risk”. Cluster 2 included 18 trials (7 players) and presented high knee extension moments peak at IC but low knee abduction and rotation moments. Players in this cluster were thus considered at “mid-risk”. Cluster 3 included 9 trials (4 players) and presented the highest knee abduction, extension moment, and external rotation moments at IC. Players in this cluster were thus considered at “high-risk” (Figure 1).

The three clusters differed in terms of average speed (*p* = 0.028, eta-squared = 0.08), with Cluster 1 (4.37 ± 0.41 m/s) moving slower than Cluster 2 (4.60 ± 0.29 m/s) and Cluster 3 (4.61 ± 0.17 m/s).

### 3.2. Field Data—Exercise Kinematics

**Figure 2:**
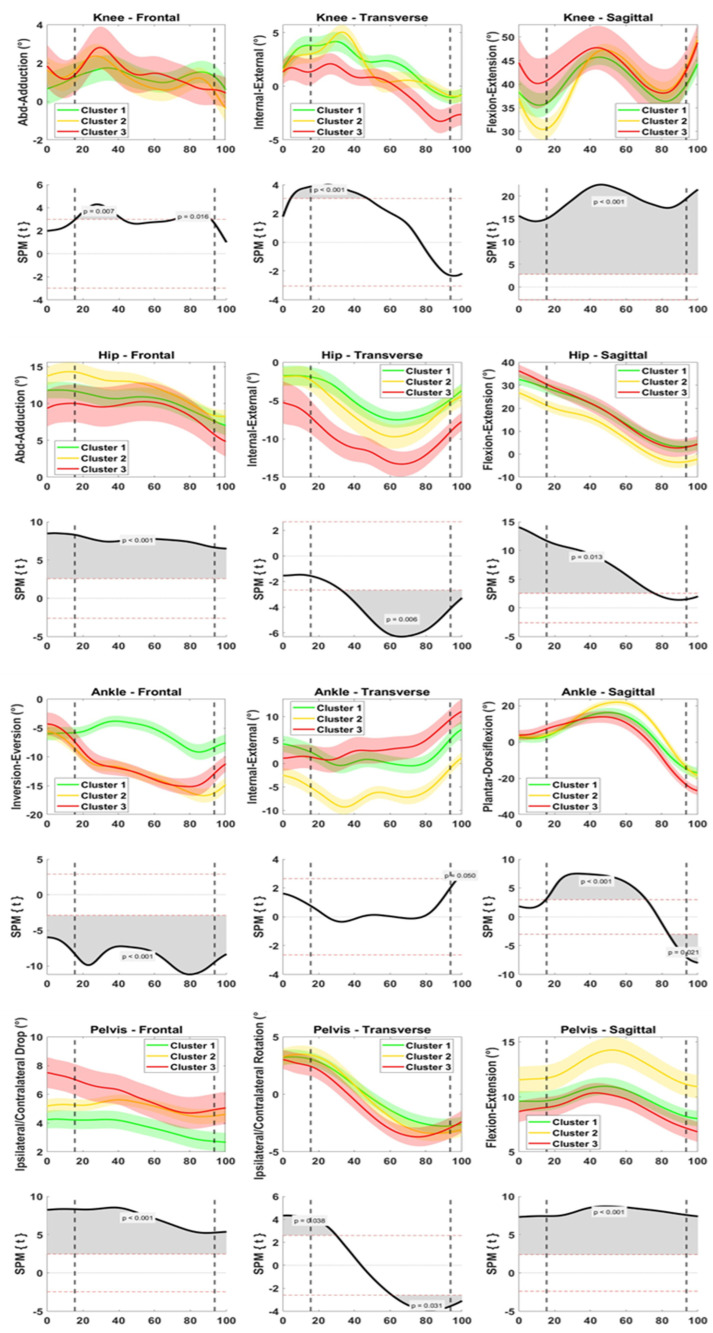
Joint kinematics of knee (1st row), hip (3rd row), ankle (5th row), and pelvis (7th row) collected on the field during change of direction exercises. Data are clustered according to the knee moments emerged in the lab (Cluster 1, green, “low-risk”; Cluster 2, yellow, “mid-risk”; Cluster 3, red, “high-risk”). Data are presented as mean and standard deviation on frontal (1st column), transverse (2nd column), and sagittal (3rd column) plane. One-way ANOVA through Statistical Parametric Mapping (SPM) was used to compare the groups (rows 2, 4, 6, 8 for knee, hip, ankle, and pelvis, respectively). Data are normalized from 50 ms prior to foot initial contact to 25 ms after toe-off. Dotted lines represent initial contact and toe-off. Positive rotations: flexion (dorsiflexion, anteversion) on sagittal plane, abduction (abduction, inversion, ipsilateral tilt) on frontal plane, and internal rotation (ipsilateral rotation) on transverse plane.

At the knee joint, differences among the clusters were mainly found in the first 40% of the time window, i.e., at IC and during weight acceptance (Figure 2). On the frontal plane, Clusters 2 and 3 exhibited greater knee abduction than Cluster 1 (*p* = 0.007). On the transverse plane, Cluster 3 exhibited lower knee internal rotation than the other clusters (*p* < 0.001). On the sagittal plane, Cluster 2 exhibited lower knee flexion than the other clusters at IC and up to peak knee flexion (*p* < 0.001).

At the hip joint frontal plane, hip abduction at IC differed among the three clusters (greatest in Cluster 2, lowest in Cluster 3, *p* < 0.001, Figure 2). On the transverse plane, Cluster 3 exhibited greater hip external rotation than the other clusters (*p* = 0.006). On the sagittal plane, Cluster 2 exhibited lower hip flexion than the other clusters up to 70% of the movement (*p* < 0.001).

At the ankle joint, differences among the clusters were found on the frontal and sagittal plane (Figure 2). On the frontal plane, Clusters 2 and 3 exhibited greater ankle eversion than Cluster 1 (*p* < 0.001). On the sagittal plane, Cluster 2 exhibited greater dorsiflexion peak (50–60% of the time window) than the other clusters (*p* < 0.001).

For the pelvis, Cluster 3 exhibited greater ipsilateral pelvic tilt (all time window, *p* < 0.001) and rotation (at IC and TO, *p* 0.031–0.038) than the other clusters. On the sagittal plane, Cluster 2 exhibited greater pelvis anteversion (*p* < 0.001).

### 3.3. Field Data—Game Kinematics

**Figure 3:**
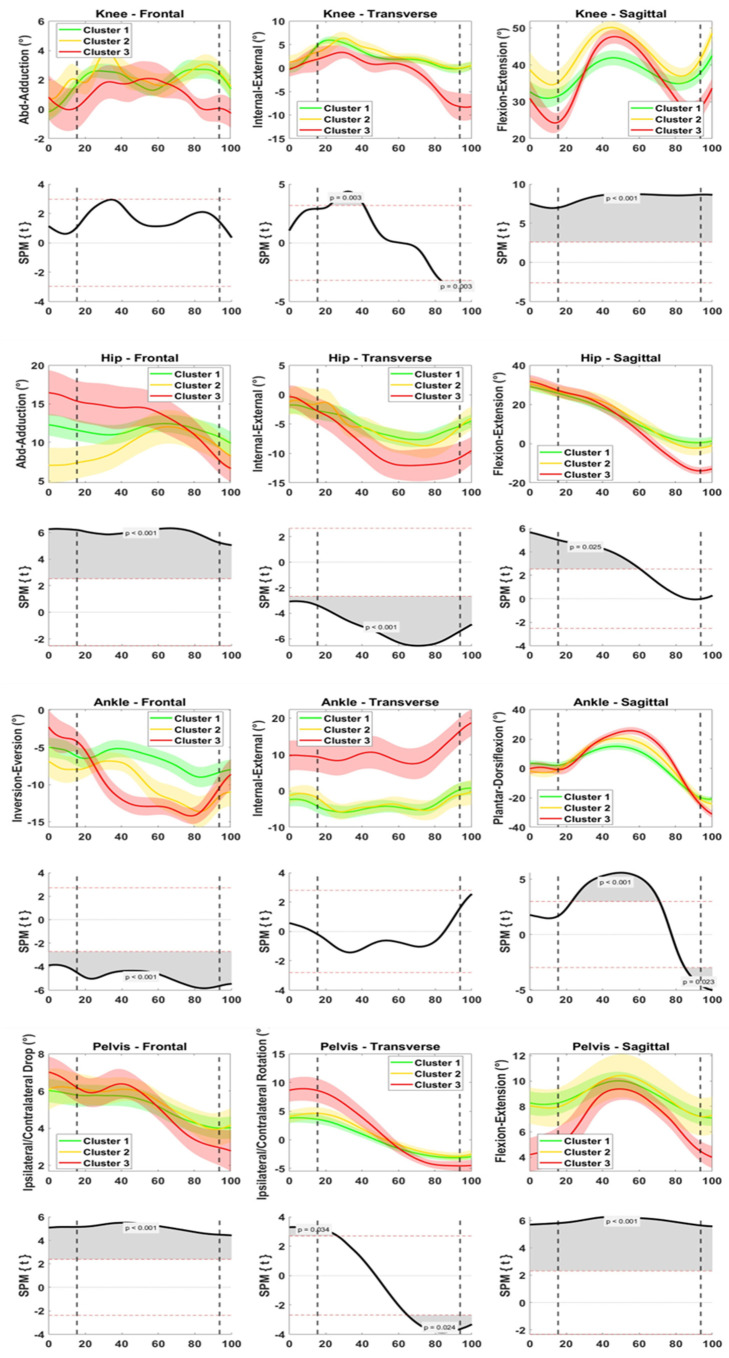
Joint kinematics of knee (1st row), hip (3rd row), ankle (5th row), and pelvis (7th row) collected on the field during change of direction in game. Data are clustered according to the knee moments emerged in the lab (Cluster 1, green, “low-risk”; Cluster 2, yellow, “mid-risk”; Cluster 3, red, “high-risk”). Data are presented as mean and standard deviation on the frontal (1st column), transverse (2nd column), and sagittal (3rd column) plane. One-way ANOVA through Statistical Parametric Mapping (SPM) was used to compare the groups (rows 2, 4, 6, 8 for knee, hip, ankle, and pelvis, respectively). Data are normalized from 50 ms prior to foot initial contact to 25 ms after toe-off. Dotted lines represent initial contact and toe-off. Positive rotations: flexion (dorsiflexion, anteversion) on sagittal plane, abduction (abduction, inversion, ipsilateral tilt) on frontal plane, and internal rotation (ipsilateral rotation) on transverse plane.

At the knee joint, differences among the clusters were found on the transverse plane after IC (20–40% of the time window) and at TO and on the sagittal plane during all the time window (Figure 3). Cluster 3 exhibited lower knee internal rotation (*p* = 0.003) and lower knee flexion than the other clusters (*p* < 0.001).

At the hip joint frontal plane, hip abduction at IC differed among the three clusters differently from the F-EX (greatest in Cluster 3, lowest in Cluster 2, *p* < 0.001, Figure 3). Cluster 3 exhibited greater hip external rotation (*p* < 0.001) and hip extension than the other clusters (*p* < 0.025).

At the ankle joint, differences among the clusters were found on the frontal and sagittal plane (Figure 3). On the frontal plane, Clusters 2 and 3 exhibited greater ankle eversion than Cluster 1 (*p* < 0.001), as for the F-EX. On the sagittal plane, Cluster 2 exhibited greater dorsiflexion peak (50–60% of the time window, *p* < 0.001) and plantarflexion peak (*p* = 0.023) than the other clusters.

At the pelvis joint, Cluster 3 exhibited greater ipsilateral pelvic tilt range of motion (*p* < 0.001) and ipsilateral rotation (at IC and TO, *p* 0.024–0.034), and lower pelvic anteversion (*p* < 0.001) than the other clusters.

Differences in speed among the three clusters were noted both during exercise and game changes of direction, while no difference in cut angle was found (Table 1).

## 4. Discussion

The present study was the first to investigate whether players considered at risk for ACL injury according to well-established risk factors collected in a laboratory environment would present an at-risk movement pattern during football-specific movements on the field. Such an analysis is crucial to understand if at-risk movement strategies are maintained in an ecological environment or, vice versa, if “safe” movement strategies are not; thus, if laboratory analysis is enough to assess a player’s level of risk for ACL injury.

The main finding of the present study was that clinically relevant differences towards ACL injury identified when cutting in the laboratory reflected at-risk movement patterns only in part when cutting in the field.

**Figure 4:**
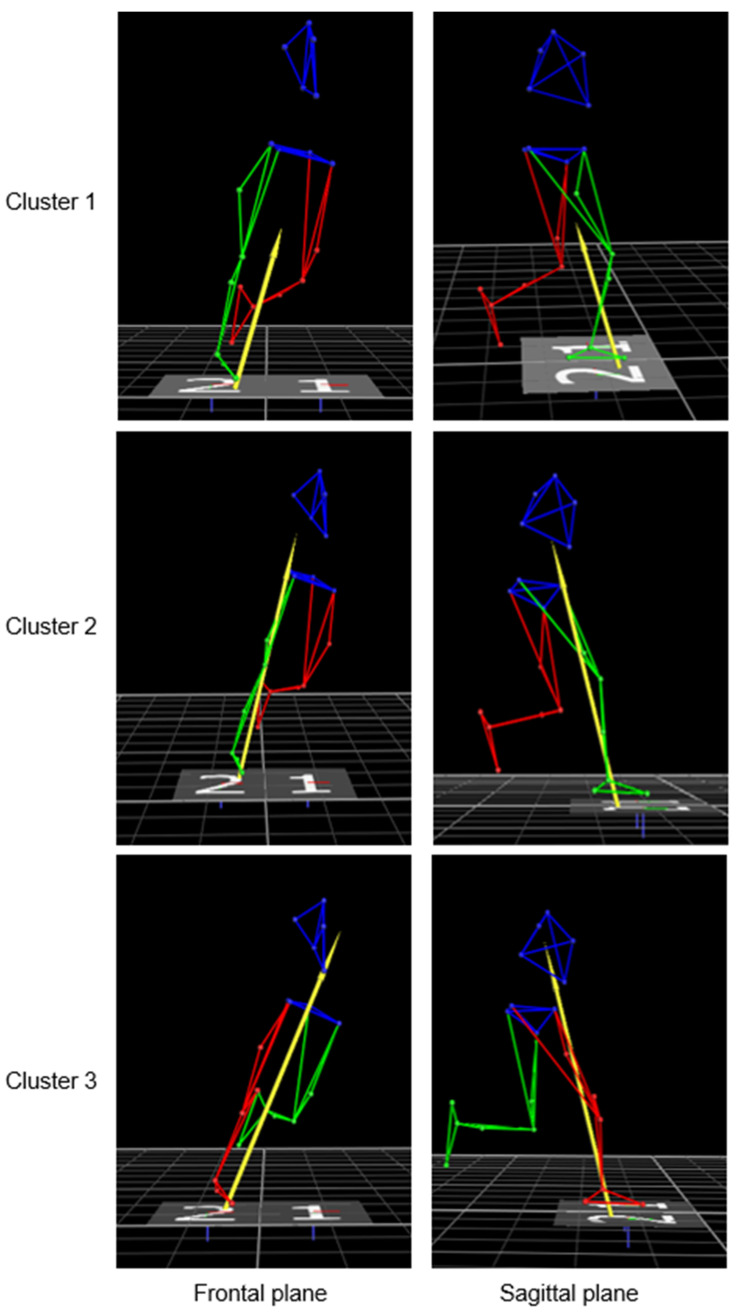
Example of trials belonging to Cluster 1 (first row), Cluster 2 (second row), and Cluster 3 (third row) from frontal (**left**) and sagittal (**right**) view. The frame represents initial contact. Cluster 1 had lowest peak knee moments in the three planes and was considered low-risk; Cluster 2 had high knee extension moment at initial contact and was considered mid-risk (note the higher magnitude and the vector close to the knee on sagittal plane); Cluster 3 had high knee abduction and knee extension moment at initial contact and was considered high-risk (note the higher magnitude and the vector close to the knee on sagittal plane and external to the knee on the frontal plane).

Three main clusters emerged based on knee joint moments captured in the lab during unanticipated 45° cutting maneuver: a “high-risk” cluster with high knee abduction and extension moments at IC; a “mid-risk” cluster with high knee extension moments at IC; and a “low-risk” cluster with the lowest knee joint moments at IC in all three planes (Figure 4). The three clusters could be highly representative of the different movement strategies adopted by the players when performing an agility maneuver in terms of knee joint loading. Despite comparable cutting angles and approaching speed, players in Cluster 2 and Cluster 3 put higher “demand” on their knee according to widely accepted risk factors for ACL injury such as knee valgus collapse [25,26] and limited knee flexion [27,28]. Previous prospective studies on female athletes belonging from different sports agreed on the increased risk for ACL injury in the presence of such biomechanical factors [24,29,30]. Therefore, by means of agglomerative clustering techniques, it was possible to identify clinically relevant optimal and suboptimal cutting strategies. DiCesare and colleagues previously adopted agglomerative clustering to identify different coordination strategies in drop vertical jump technique in a cohort of 780 female adolescent athletes [22]. Such a data mining approach allows to depict hidden movement dynamics and cluster players according to biomechanical features belonging from multiple waveforms (in this case, knee moments in the three planes).

The present study identified clinically relevant differences between the three clusters during both the field exercise and game cuts. In particular, Cluster 3 had worse biomechanical patterns with players who presented greater knee abduction, hip external rotation, ankle eversion, and pelvis tilt in the field exercise compared to the other two clusters [4,31]. Thus, the main differences of this Cluster 3 from Cluster 1 (and, less evidently, from Cluster 2) were on frontal/transverse plane in all joints. In the field game, less evident differences at knee level emerged among the clusters: peak values were more similar in magnitude, whole waveforms were more similar in shapes, and overall larger data dispersion was noted (Figure 3). As with the field exercise, greater hip external rotation, pelvis ipsilateral tilt and rotation, and ankle eversion were found in Cluster 3 during the game. The frontal and transverse plane kinematics of Cluster 3 players is in line with previous literature on at-risk kinematics [24,31,32,33] and recalls the kinematics at the time of ACL injury reported in elite male and female football players, respectively [4,25].

Cluster 2 players showed a stiffer kinematic strategy compared to the other clusters with less hip and knee flexion at initial contact, more pelvic anteversion, and ankle dorsiflexion in the field exercise (Figure 2). Thus, the main differences from Cluster 1 (and, less evidently, from Cluster 3) were found on the sagittal plane. In the field game, a stiffer strategy compared to the other two clusters was not maintained. A stiffer kinematics strategy is commonly found as a predictor of ACL injury risk in prospective studies assessing different movement tasks and sports [28,34,35]. Indeed, the achievement of a good range of lower limb motion is a widespread target of ACL injury prevention programs [32,36,37]. The Cluster 1 players did not exhibit knee abduction, showed limited rotations on transverse and frontal planes at all joints, and smooth (average) sagittal plane angle.

Thus, the differences among Clusters identified in the lab appeared to be preserved in the field: greater knee abduction moment in the lab was associated with greater frontal plane angles in the field and greater knee extensor moment in the lab was associated with stiffer lower limb kinematics. Moreover, low knee joint moments captured in the lab (Cluster 1, low-risk) were associated with low frontal/transverse plane kinematics and smooth sagittal plane flexion. However, the differences among the clusters were less evident moving from the field exercise to the field game, especially at the knee level and on the sagittal plane for all joints. These aspects suggest that in the game-resembling condition, the ACL injury risk assumption based on laboratory-based biomechanics should be slightly adjusted according to the complexity and unpredictability of the environment. On the field, the assessment of the knee kinematics alone could be not sufficient enough to draw inferences on the ACL injury risk.

Previous research identified biomechanical differences between the kinematics acquired in the lab and the field [13]. As advocated in the recent literature, the assessment of athletes’ motion in their own sport-specific environment is becoming fundamental to further improve the prevention programs and thus reduce the risk of ACL injury [5,38,39]. This is particularly true in a quickly emerging sport such as young female football. The adoption of a field-based clustering paradigm that takes into consideration the whole-body motion instead of a single joint movement pattern is deemed important in ACL injury prevention research. For example, recent studies are adopting vector coding technique to identify intra- and inter-joint coordination for trunk, hip, knee, and ankle, and link it to the risk of sustaining an ACL injury [38,40,41,42]. Data mining approaches based on waveform features such as the one adopted in the present study might facilitate this process, reducing the number of informative features while continuing to account for the task complexity [22,42,43,44,45].

From a practical point of view, football coaches might add the quantitative information of each player to personalize their screening activity towards ACL injury prevention. Despite ACL injury prevention programs which have sensibly improved over the years in young footballers (e.g., the FIFA 11+ [46]), such technologies might support dealing with the complexity of the football environment interactions [47]. For example, Cluster 1 players had the largest sample size in the present study cohort: the kinematics (and kinetics) of these players might be interpreted as a benchmark for the female footballers, with a protective motor strategy towards the ACL injury, and used as an example for more at-risk players [48].

The present study has some limitations. Cut angle in the field was in a wider range than in the laboratory (30–60° vs. 40–50°). Although this means that the cut maneuver in the lab could have been different from the one on the field, this was not relevant for cluster comparison, which was based on data with the same cut angle (*p* > 0.05, Table 1). This was performed to include adequate F-GAME samples and avoid loss of statistical power. The authors believe that providing data from a wider angle range leads to a more comprehensive interpretation and future use of on-field data, not limiting the inter/intra-subject motion variability recorded. Moreover, previous studies identified a similar risk band for trials performed with a cut angle lower than 60° [18]. Cutting data performed during field exercise and small-sided games had never been assessed in the literature. Thus, it is hard to perform a direct comparison of the quantitative kinematics acquired on the field in the present study. However, previous studies assessing ACL injury risk patterns at initial contact and injury frame in football based on video analysis have been used as a reference for at-risk kinematics [4,25]. It was not possible to account for the presence of teammates, ball, and opponents from a kinematical point of view. Future studies might selectively account for the kinematical changes and injury risk pattern occurrence in the presence of single football-specific elements. Despite the wearable sensor system used in the present study which has been validated against the gold standard for the assessment of high-dynamics tasks, caution should be adopted in the interpretation of transverse plane angles at the knee and ankle, as previously suggested [49,50]. Lastly, the present study cohort was made of healthy players only. Therefore, it was not possible to account for the kinematics (and kinetics) of ACL-reconstructed players. Such an analysis might offer precious insights on the biomechanical differences that should be taken into account both in prevention and rehabilitation training and assessment programs.

## 5. Conclusions

In this unique study, we investigated one cohort of young female football players and compared their movements in the laboratory and in the field. In the lab, we found a “high-risk” cluster with high knee abduction and knee extension moments at IC; a “mid-risk” cluster with high knee extension moments at IC, and a “low-risk” cluster with the lowest knee joint moments in all three planes. Clinically relevant differences towards ACL injury risk identified in the lab were preserved on the field but differences among clusters were less evident as environmental complexity increased. The high knee external rotation moments found in the “high-risk” players in the lab were reflected in the exercise and field game condition with high knee external rotation angles. Moreover, these players showed the lowest knee flexion angles in the field game. In the field game, the “low-risk” players showed similar kinematic patterns as the high-risk players. Therefore, in-lab injury risk screening may lack ecological validity. A field-based paradigm should be included in the identification of players at risk for ACL injury, considering a whole-body motion instead of knee joint moments only.

## Figures and Tables

**Table 1:** Maximum speed and cut angle of the movement tasks performed on the field during exercise and game activities.

Cluster	Cluster 1 “Low-Risk”	Cluster 1 “Mid-Risk”	Cluster 3 “High-Risk”	*p*-Value	Eta-Squared
Exercise					
No. of trials	48	44	19		
Speed (m/s)	3.5 ± 0.9	3.2 ± 0.8	2.7 ± 0.7	0.003	0.11
Angle (°)	32.3 ± 14.2	40.4 ± 18.6	38.5 ± 20.6	n.s.	0.05
Game					
No. of trials	45	22	14		
Speed (m/s)	3.7 ± 1.0	3.0 ± 0.8	3.2 ± 0.7	0.016	0.10
Angle (°)	29.0 ± 15.0	33.7 ± 17.0	29.6 ± 15.8	n.s.	0.02

Note: The clustering of players was based on the knee kinetics collected in the lab; n.s. = non-significant differences.

## Data Availability

Not applicable.

## Supplementary Materials

The following supporting information can be downloaded at: https://www.mdpi.com/article/10.3390/s23042176/s1.

## References

[1] Agel J., Rockwood T., Klossner D. (2016). Collegiate ACL Injury Rates Across 15 Sports: National Collegiate Athletic Association Injury Surveillance System Data Update (2004–2005 Through 2012–2013). Clin. J. Sport Med. Off. J. Can. Acad. Sport Med..

[2] Aït Si Selmi T., Fithian D., Neyret P. (2006). The Evolution of Osteoarthritis in 103 Patients with ACL Reconstruction at 17 Years Follow-Up. Knee.

[3] Brophy R.H., Stepan J.G., Silvers H.J., Mandelbaum B.R. (2015). Defending Puts the Anterior Cruciate Ligament at Risk During Soccer: A Gender-Based Analysis. Sport. Health Multidiscip. Approach.

[4] Lucarno S., Zago M., Buckthorpe M., Grassi A., Tosarelli F., Smith R., Della Villa F. (2021). Systematic Video Analysis of Anterior Cruciate Ligament Injuries in Professional Female Soccer Players. Am. J. Sport. Med..

[5] Bolt R., Heuvelmans P., Benjaminse A., Robinson M.A., Gokeler A. (2021). An Ecological Dynamics Approach to ACL Injury Risk Research: A Current Opinion. Sport. Biomech..

[6] Gokeler A., Benjaminse A., Della Villa F., Tosarelli F., Verhagen E., Baumeister J. (2021). Anterior Cruciate Ligament Injury Mechanisms through a Neurocognition Lens: Implications for Injury Screening. BMJ Open Sport Exerc. Med..

[7] McLean S.G., Huang X., van den Bogert A.J. (2005). Association between Lower Extremity Posture at Contact and Peak Knee Valgus Moment during Sidestepping: Implications for ACL Injury. Clin. Biomech..

[8] Sigward S.M., Powers C.M. (2006). The Influence of Gender on Knee Kinematics, Kinetics and Muscle Activation Patterns during Side-Step Cutting. Clin. Biomech..

[9] Cortes N., Blount E., Ringleb S., Onate J.A. (2011). Soccer-Specific Video Simulation for Improving Movement Assessment. Sport. Biomech..

[10] Lee M.J.C., Lloyd D.G., Lay B.S., Bourke P.D., Alderson J.A. (2013). Effects of Different Visual Stimuli on Postures and Knee Moments during Sidestepping. Med. Sci. Sport. Exerc..

[11] McGuckian T.B., Cole M.H., Pepping G.-J. (2018). A Systematic Review of the Technology-Based Assessment of Visual Perception and Exploration Behaviour in Association Football. J. Sport. Sci..

[12] Krosshaug T., Steffen K., Kristianslund E., Nilstad A., Mok K.-M., Myklebust G., Andersen T.E., Holme I., Engebretsen L., Bahr R. (2016). The Vertical Drop Jump Is a Poor Screening Test for ACL Injuries in Female Elite Soccer and Handball Players: A Prospective Cohort Study of 710 Athletes. Am. J. Sport. Med..

[13] Di Paolo S., Nijmeijer E., Bragonzoni L., Dingshoff E., Gokeler A., Benjaminse A. (2022). Comparing Lab and Field Agility Kinematics in Young Talented Female Football Players: Implications for ACL Injury Prevention. Eur. J. Sport Sci..

[14] Newell K.M., Van Emmerik R.E.A., McDonald P.V. (1989). Biomechanical Constraints and Action Theory. Hum. Mov. Sci..

[15] Vicon Motion Systems Vicon Nexus User Guide. https://docs.vicon.com/display/Nexus212/Vicon+Nexus+User+Guide/.

[16] Kadaba M.P., Ramakrishnan H.K., Wooten M.E., Gainey J., Gorton G., Cochran G.V.B. (1989). Repeatability of Kinematic, Kinetic, and EMG Data in Normal Adult Gait. J. Orthop. Res..

[17] McGinley J.L., Baker R., Wolfe R., Morris M.E. (2009). The Reliability of Three-Dimensional Kinematic Gait Measurements: A Systematic Review. Gait Posture.

[18] Dos’Santos T., Thomas C., Jones P.A. (2021). The Effect of Angle on Change of Direction Biomechanics: Comparison and Inter-Task Relationships. J. Sport. Sci..

[19] Koga H., Nakamae A., Shima Y., Iwasa J., Myklebust G., Engebretsen L., Bahr R., Krosshaug T. (2010). Mechanisms for Noncontact Anterior Cruciate Ligament Injuries: Knee Joint Kinematics in 10 Injury Situations from Female Team Handball and Basketball. Am. J. Sport. Med..

[20] Hewett T.E., Myer G.D., Ford K.R., Heidt R.S., Colosimo A.J., McLean S.G., Van Den Bogert A.J., Paterno M.V., Succop P. (2005). Biomechanical Measures of Neuromuscular Control and Valgus Loading of the Knee Predict Anterior Cruciate Ligament Injury Risk in Female Athletes: A Prospective Study. Am. J. Sport. Med..

[21] Myer G.D., Ford K.R., Di Stasi S.L., Foss K.D.B., Micheli L.J., Hewett T.E. (2015). High Knee Abduction Moments Are Common Risk Factors for Patellofemoral Pain (PFP) and Anterior Cruciate Ligament (ACL) Injury in Girls: Is PFP Itself a Predictor for Subsequent ACL Injury?. Br. J. Sport. Med..

[22] Dicesare C.A., Minai A.A., Riley M.A., Ford K.R., Hewett T.E., Myer G.D. (2020). Distinct Coordination Strategies Associated with the Drop Vertical Jump Task. Med. Sci. Sport. Exerc..

[23] Sigurðsson H.B., Briem K. (2019). Cluster Analysis Successfully Identifies Clinically Meaningful Knee Valgus Moment Patterns: Frequency of Early Peaks Reflects Sex-Specific ACL Injury Incidence. J. Exp. Orthop..

[24] Sigurðsson H.B., Karlsson J., Snyder-Mackler L., Briem K. (2021). Kinematics Observed during ACL Injury Are Associated with Large Early Peak Knee Abduction Moments during a Change of Direction Task in Healthy Adolescents. J. Orthop. Res..

[25] Della Villa F., Buckthorpe M., Grassi A., Nabiuzzi A., Tosarelli F., Zaffagnini S., Della Villa S. (2020). Systematic Video Analysis of ACL Injuries in Professional Male Football (Soccer): Injury Mechanisms, Situational Patterns and Biomechanics Study on 134 Consecutive Cases. Br. J. Sport. Med..

[26] Dix C., Arundale A., Silvers-Granelli H., Marmon A., Zarzycki R., Snyder-Mackler L. (2020). Biomechanical Measures during Two Sport-Specific Tasks Differentiate between Soccer Players Who Go on to Anterior Cruciate Ligament Injury and Those Who Do Not: A Prospective Cohort Analysis. Int. J. Sport. Phys. Ther..

[27] Donelon T.A., Dos’Santos T., Pitchers G., Brown M., Jones P.A. (2020). Biomechanical Determinants of Knee Joint Loads Associated with Increased Anterior Cruciate Ligament Loading During Cutting: A Systematic Review and Technical Framework. Sport. Med.-Open.

[28] Leppänen M., Pasanen K., Krosshaug T., Kannus P., Vasankari T., Kujala U.M., Bahr R., Perttunen J., Parkkari J. (2017). Sagittal Plane Hip, Knee, and Ankle Biomechanics and the Risk of Anterior Cruciate Ligament Injury: A Prospective Study. Orthop. J. Sport. Med..

[29] Bates N.A., Myer G.D., Hale R.F., Schilaty N.D., Hewett T.E. (2020). Prospective Frontal Plane Angles Used to Predict ACL Strain and Identify Those at High Risk for Sports-Related ACL Injury. Orthop. J. Sport. Med..

[30] Hewett T.E., Myer G.D., Kiefer A.W., Ford K.R. (2015). Longitudinal Increases in Knee Abduction Moments in Females during Adolescent Growth. Med. Sci. Sport. Exerc..

[31] Di Paolo S., Bragonzoni L., Della Villa F., Grassi A., Zaffagnini S. (2022). Do Healthy Athletes Exhibit At-Risk Biomechanics for Anterior Cruciate Ligament Injury during Pivoting Movements?. Sport. Biomech..

[32] Alentorn-Geli E., Myer G.D., Silvers H.J., Samitier G., Romero D., Lázaro-Haro C., Cugat R. (2009). Prevention of Non-Contact Anterior Cruciate Ligament Injuries in Soccer Players. Part 1: Mechanisms of Injury and Underlying Risk Factors. Knee Surg. Sport. Traumatol. Arthrosc..

[33] Shultz S.J., Schmitz R.J. (2009). Effects of Transverse and Frontal Plane Knee Laxity on Hip and Knee Neuromechanics during Drop Landings. Am. J. Sport. Med..

[34] Leppänen M., Pasanen K., Kujala U.M., Vasankari T., Kannus P., Äyrämö S., Krosshaug T., Bahr R., Avela J., Perttunen J. (2017). Stiff Landings Are Associated With Increased ACL Injury Risk in Young Female Basketball and Floorball Players. Am. J. Sport. Med..

[35] Paterno M.V., Kiefer A.W., Bonnette S., Riley M.A., Schmitt L.C., Ford K.R., Myer G.D., Shockley K., Hewett T.E. (2015). Prospectively Identified Deficits in Sagittal Plane Hip–Ankle Coordination in Female Athletes Who Sustain a Second Anterior Cruciate Ligament Injury after Anterior Cruciate Ligament Reconstruction and Return to Sport. Clin. Biomech..

[36] Silvers-Granelli H.J., Bizzini M., Arundale A., Mandelbaum B.R., Snyder-Mackler L. (2017). Does the FIFA 11+ Injury Prevention Program Reduce the Incidence of ACL Injury in Male Soccer Players?. Clin. Orthop..

[37] Slauterbeck J.R., Choquette R., Tourville T.W., Krug M., Mandelbaum B.R., Vacek P., Beynnon B.D. (2019). Implementation of the FIFA 11+ Injury Prevention Program by High School Athletic Teams Did Not Reduce Lower Extremity Injuries: A Cluster Randomized Controlled Trial. Am. J. Sport. Med..

[38] Heidarnia E., Letafatkar A., Khaleghi-Tazji M., Grooms D.R. (2022). Comparing the Effect of a Simulated Defender and Dual-Task on Lower Limb Coordination and Variability during a Side-Cut in Basketball Players with and without Anterior Cruciate Ligament Injury. J. Biomech..

[39] Olivares-Jabalera J., Fílter-Ruger A., Dos’Santos T., Afonso J., Della Villa F., Morente-Sánchez J., Soto-Hermoso V.M., Requena B. (2021). Exercise-Based Training Strategies to Reduce the Incidence or Mitigate the Risk Factors of Anterior Cruciate Ligament Injury in Adult Football (Soccer) Players: A Systematic Review. Int. J. Environ. Res. Public. Health.

[40] Davis K., Williams J.L., Sanford B.A., Zucker-Levin A. (2019). Assessing Lower Extremity Coordination and Coordination Variability in Individuals with Anterior Cruciate Ligament Reconstruction during Walking. Gait Posture.

[41] Weir G., van Emmerik R., Jewell C., Hamill J. (2019). Coordination and Variability during Anticipated and Unanticipated Sidestepping. Gait Posture.

[42] Shao E., Mei Q., Ye J., Ugbolue U.C., Chen C., Gu Y. (2022). Predicting Coordination Variability of Selected Lower Extremity Couplings during a Cutting Movement: An Investigation of Deep Neural Networks with the LSTM Structure. Bioengineering.

[43] Di Paolo S., Santillozzi F., Zinno R., Barone G., Bragonzoni L. (2022). On-Field Biomechanical Assessment of High and Low Dive in Competitive 16-Year-Old Goalkeepers through Wearable Sensors and Principal Component Analysis. Sensors.

[44] Federolf P.A. (2016). A Novel Approach to Study Human Posture Control: “Principal Movements” Obtained from a Principal Component Analysis of Kinematic Marker Data. J. Biomech..

[45] Smeets A., Verheul J., Vanrenterghem J., Staes F., Vandenneucker H., Claes S., Verschueren S. (2020). Single-Joint and Whole-Body Movement Changes in Anterior Cruciate Ligament Athletes Returning to Sport. Med. Sci. Sport. Exerc..

[46] FIFA Medical Assessment and Research Centre Fifa11+Manual. https://www.fifa.com/.

[47] Gokeler A., Grassi A., Hoogeslag R., van Houten A., Lehman T., Bolling C., Buckthorpe M., Norte G., Benjaminse A., Heuvelmans P. (2022). Return to Sports after ACL Injury 5 Years from Now: 10 Things We Must Do. J. Exp. Orthop..

[48] Nijmeijer E.M., Elferink-Gemser M.T., Otten E., Benjaminse A. (2022). Optimal and Suboptimal Video Instructions Change Movement Execution in Young Talented Basketball Players. Int. J. Sport. Sci. Coach..

[49] Di Paolo S., Lopomo N.F., Della Villa F., Paolini G., Figari G., Bragonzoni L., Grassi A., Zaffagnini S. (2021). Rehabilitation and Return to Sport Assessment after Anterior Cruciate Ligament Injury: Quantifying Joint Kinematics during Complex High-Speed Tasks through Wearable Sensors. Sensors.

[50] van der Kruk E., Reijne M.M. (2018). Accuracy of Human Motion Capture Systems for Sport Applications; State-of-the-Art Review. Eur. J. Sport Sci..

