# Supplementary material for: Definition of High-Risk Motion Patterns for Female ACL Injury Based on Football-Specific Field Data: A Wearable Sensors Plus Data Mining Approach
Source: Sensors (Basel). 2023 Feb 15;23(4):2176. doi: 10.3390/s23042176 (PMC9961558; doi:10.3390/s23042176)
Supplement: Supplementary file 1 [file sensors-23-02176-s001.zip › sensors-2194909-supplementary.pdf]

Laboratory

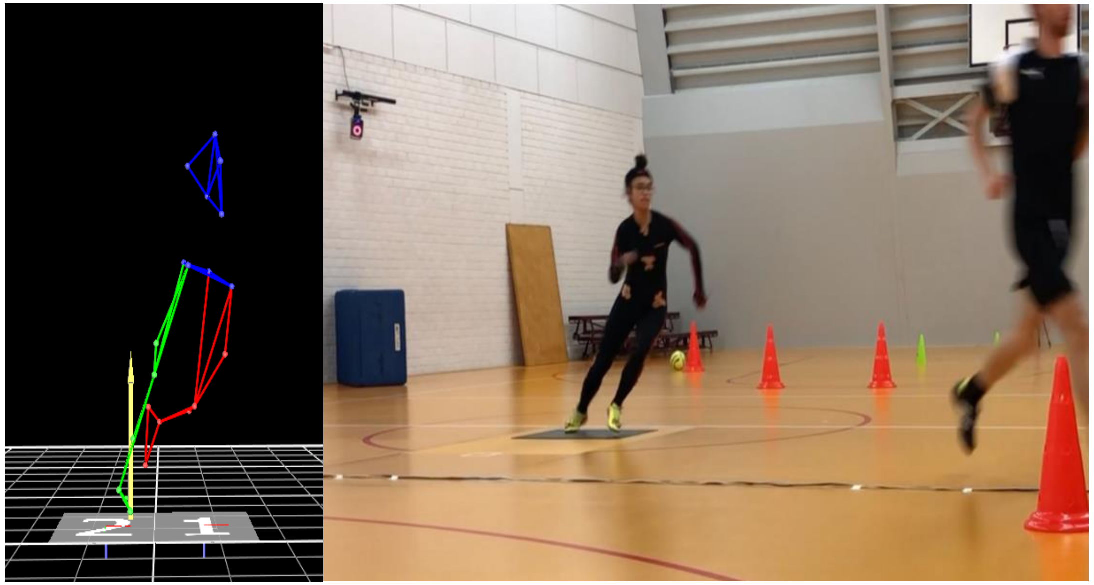

System
- Eight optoelectronic cameras (Vicon Motion Systems)
- Two force plates
- Sixteen markers on pelvis and lower limbs (Vicon Plug-in-Gait lower body model)
- Five additional trunk markers on the sternum, clavicle, C7, T10 and right scapula

Data collection
- Knee joint moments (frontal, transverse, and sagittal plane) from unanticipated changes of direction at 40-50°

Data processing
- Hierarchical agglomerative clustering (Ward's linkage method) on knee joint moments through discrete wavelet transform with the Haar wavelet

➢ **Players split in three clusters:** low-risk (lowest moments), mid-risk (high sagittal and low frontal/transverse plane moments), high-risk (highest moments)

Football (soccer) field

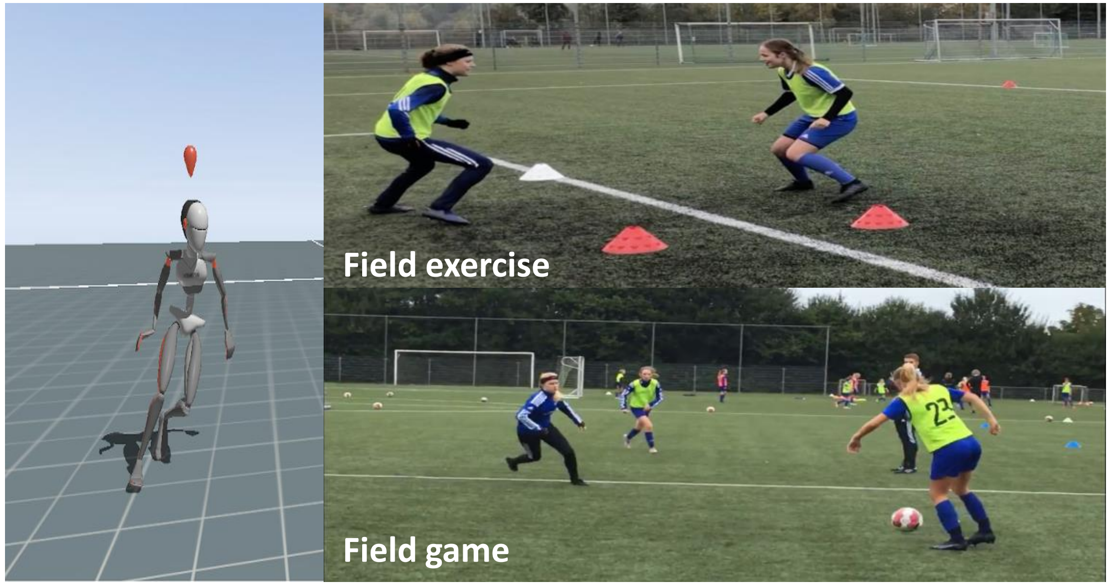

System
- Seventeen inertial measurement units (Link, Xsens)
- Full body configuration (upper limbs, head, trunk, pelvis, lower limbs)

Data collection
- Football-specific exercises with teammates, opponents, and ball (F-EX) and football game (F-GAME)
- Pelvis, hip, knee, ankle joint kinematics (frontal, transverse, and sagittal plane) from unanticipated changes of direction at 30-60°

Data processing
- One-way ANOVA with hierarchical two-level random effect model based on clusters obtained from knee joint moments in laboratory

Please refer to Di Paolo S. et al., EJSS, 2022 (DOI: 10.1080/17461391.2022.2064771) for further details on data collection methodology
